# Recursive algorithms for phylogenetic tree counting

**DOI:** 10.1186/1748-7188-8-26

**Published:** 2013-10-28

**Authors:** Alexandra Gavryushkina, David Welch, Alexei J Drummond

**Affiliations:** 1 Department of Computer Science, The University of Auckland, Auckland, New Zealand; 2 Allan Wilson Centre for Molecular Ecology and Evolution, University of Auckland, Auckland, New Zealand

**Keywords:** Ranked tree, Constraint tree, Resolution, Counting trees, Dynamic algorithms, Bayesian tree prior, Phylogenetics

## Abstract

**Background:**

In Bayesian phylogenetic inference we are interested in distributions over a space of trees. The number of trees in a tree space is an important characteristic of the space and is useful for specifying prior distributions. When all samples come from the same time point and no prior information available on divergence times, the tree counting problem is easy. However, when fossil evidence is used in the inference to constrain the tree or data are sampled serially, new tree spaces arise and counting the number of trees is more difficult.

**Results:**

We describe an algorithm that is polynomial in the number of sampled individuals for counting of resolutions of a constraint tree assuming that the number of constraints is fixed. We generalise this algorithm to counting resolutions of a fully ranked constraint tree. We describe a quadratic algorithm for counting the number of possible fully ranked trees on *n* sampled individuals. We introduce a new type of tree, called a fully ranked tree with sampled ancestors, and describe a cubic time algorithm for counting the number of such trees on *n* sampled individuals.

**Conclusions:**

These algorithms should be employed for Bayesian Markov chain Monte Carlo inference when fossil data are included or data are serially sampled.

## Background

A phylogenetic tree is the common object of interest in many areas of biological science. The tree represents the ancestral relationships between a group of individuals. Given molecular sequence data sampled from a group of organisms it is possible to infer the historical relationships between these organisms using a statistical model of molecular evolution. At present, Bayesian Markov chain Monte Carlo (MCMC) methods are the dominant inferential tool for inferring molecular phylogenies [1].

It is a recent trend to include fossil evidence into the inference to obtain absolute estimates of divergence times [2,3]. Fossils may restrict the age of the most resent common ancestor of a subgroup of individuals. This imposes a constraint on the tree topology (the discrete component of a genealogy) and therefore reduces the space of allowable genealogies.

Another trend in phylogenetic analyses is serial (or heterochronous) sampling in which molecular data is obtained from significantly different time points and analysed together. This type of data arises most frequently with ancient DNA and rapidly evolving pathogens [4]‐[6]. In this case tip dates become a part of the genealogy.

Including serially sampled or fossil data modifies or restricts the shape of a phylogenetic tree. Little has been done to describe and classify these modified trees. In this paper, we aim to explore the new spaces formed by these trees.

A genealogy consists of discrete and continuous components — the tree topology and the divergence times. The tree topologies form a finite tree space when the number of tips is bounded. An important characteristic of this space is the number of trees in it and we aim to find an efficient way to calculate this number.

In the case that fossil data restricts the tree topology, counting the number of trees that satisfy the imposed constraints reveals how much the constraints reduce the tree space.

The number of trees arises as a constant in tree prior distributions. Typically we model the distribution of tree topologies as independent of the distribution of divergence times. The density function of the distribution of genealogies is then a product of the density function for the divergence times and the distribution function for tree topologies. A common prior on tree topologies is uniform over all allowable topologies so the distribution function is a constant that is equal to one over the number of tree topologies. When inferring tree topologies using Bayesian MCMC methods, we do not usually need to know this constant but in some cases, as described below, the absolute value of the prior distribution is of interest and the constant has to be calculated.

When fossils are used to restrict the age of internal nodes, the tree prior should accurately account for this fact. Heled and Drummond [3] introduced a natural approach for tree prior specification when fossil evidence is employed in the inference. Their method requires counting of ranked phylogenetic trees that obey a number of constraints that arise from including the fossil evidence. The construction requires calculation of the marginal density for the time of the calibration node, the node representing the most recent common ancestor of a clade which may or may not be monophyletic. For a particular location of the calibration node, or particular constraints on the tree topology, the marginal density function is the marginal density function for the divergence times weighted by the number of trees satisfying the constraints. In this case, the weight constants do not cancel in the MCMC scheme and therefore have to be calculated.

Tree counting has a long history. For phylogenetic trees, the counting problem is to find the number of all possible trees on *n* leaves. For some types of phylogenetic tree, there are known closed form solutions to this problem. For other types, only recursive equations have been derived. In this paper, we consider only rooted trees.

A survey of results on counting different types of rooted trees is presented in [7] where trees with different combinations of the following properties are considered: trees are either labeled (only leaves are labeled) or unlabeled, ranked or non‐ranked, and bifurcating or multifurcating. The results presented in the survey can also be found in [6,8,9].

In [10], Griffiths considered unlabeled, non‐ranked rooted trees such that interior nodes can have one child or more and the root has at least two children. Using generating functions, he derived recursive equations for counting the number of all possible such trees on *n* leaves with *s* interior nodes. In [11], Felsenstein considered partially labeled trees, i.e., a tree in which all the leaves are labeled and some interior nodes also may be labeled. He derived the recursive equations for counting the number of rooted, non‐ranked, partially labeled trees with *n* labeled nodes.

In this paper, we consider a number of counting problems for different classes of phylogenetic trees. First, we describe an effective way of counting the number of all possible fully ranked trees on *n* leaves, that is, trees on *n* leaves in which all internal and leaf nodes are ranked.

Second, we find the number of bifurcating trees that resolve a given multifurcating tree with *n* leaves. We give a solution to this problem for rooted, ranked, labeled trees and generalise the algorithm to count resolutions to fully ranked trees.

Finally, we introduce and formally describe a new type of phylogenetic tree and describe an algorithm for counting the number of all such trees on *n* leaves. This type of tree is important when we have a serial sample and sampled individuals can be direct ancestors of later sampled individuals. When the population size is small or the fraction of individuals sampled from the population is large, this type of tree should be included in the inference [12,13].

## Serial sampling

We mainly follow the terminology from [9] for the definitions of phylogenetic trees. A tree is a finite connected undirected graph with no cycles. A rooted tree is a tree with a single node *ρ* designated as a root. Every rooted tree *T*=(*V*,*E*,*ρ*) imposes a partial order on *V* that is defined as follows: *v*_1_≤_*T*_*v*_2_ if a unique simple path from the root to *v*_2_ passes through *v*_1_. So the root is the smallest element. If *v*_1_≤_*T*_*v*_2_ then we say that *v*_1_ is an ancestor of *v*_2_ and *v*_2_ is a descendant of *v*_1_. A node in a rooted tree is called interior if it has descendants and a leaf if it has no descendants. The root is considered interior. Denote $\overset{\circ }{V}$ the set of interior nodes of *T*. A node *u* is a parent of a node *v* and *v* is a child of *u* if *v*<_*T*_*u* and there is no *w*∈*V* such that *v*<_*T*_*w*<_*T*_*u*. A rooted tree is called binary if every interior node has exactly two children. It is called weakly binary if every interior node has at most two children. We have chosen this terminology to fit with the usage of “binary” in the phylogenetics literature which may not agree with that in other literatures.

Let *X* be a finite non‐empty set of labels. *A phylogenetic X‐tree* is a pair T=(T,ϕ), where *T* is a tree and *ϕ* is a bijection from *X* onto the set of leaves of *T* (we may omit *X* and say “tree” instead of “*X*‐tree” if the set of labels is not specified). The tree *T* is called an underlying tree or a shape of the phylogenetic tree  and *ϕ* is a labeling function. If the underlying tree of  is rooted then  is called *a rooted phylogenetic tree*. In what follows, we consider only rooted trees unless explicitly stated otherwise. A phylogenetic tree is *binary (weak binary)* if its underlying tree is binary (weak binary). *A ranked phylogenetic tree* is a pair (T,h), where  is a rooted phylogenetic tree and *h* is an injective function (*ranking function*) from the set $\overset{\circ }{V}$ into the set $\left\{1,\ldots ,|\overset{\circ }{V}|\right\}$ such that *v*_1_≤_*T*_*v*_2_ implies *h*(*v*_1_)≤*h*(*v*_2_) for every $v_{1},v_{2}\in \overset{\circ }{V}$. In other words, there is a linear order on the interior nodes of *T* that is consistent with the partial order of *T*.

### Definition 1

***A ranked X‐tree*** *is a binary ranked phylogenetic X‐tree.*

**Figure 1:**
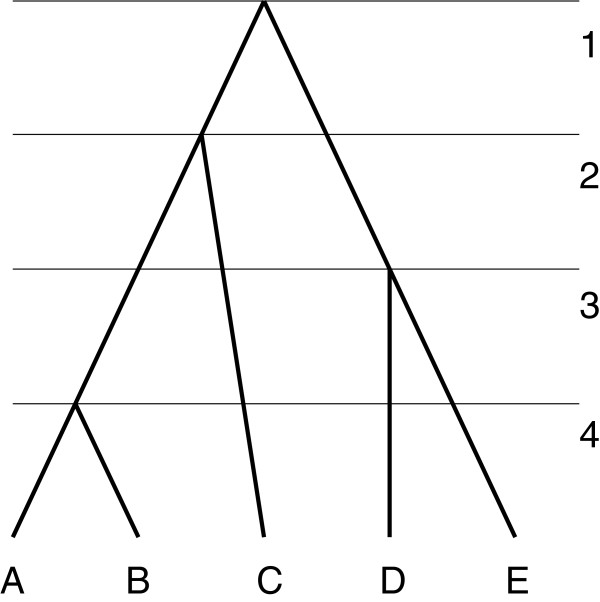
**Ranked tree.** Ranked *X*‐tree, *X*={*A*,*B*,*C*,*D*,*E*}. The numbers on the right are values of the ranking function.

An example of a ranked tree is given in Figure 1.

In biology, a phylogenetic tree represents the evolutionary history of a collection of sampled individuals. The collection of individuals is represented by the set *X*. The root of the tree is the most recent common ancestor of *X* and interior nodes are bifurcation events. The ranking function represents the time order of the bifurcation events. A general problem in evolutionary biology is how to reconstruct the phylogenetic tree from sequence data obtained from sampled individuals. Tackling this problem in a Bayesian framework may require counting the number of all possible histories on a sample of individuals.

When all individuals are sampled at the same time (as in Figure 1) counting tree problem has a simple solution.

Let *X* be a fixed label set such that |*X*|=*n*. The number of all ranked *X*‐trees up to isomorphism is

$$R(n)=\frac{n!(n-1)!}{2^{n-1}}$$

This formula has been derived by many authors. Proofs can be found in [6,7], or [9]. The letter *R* in the equation comes from the word “ranked”.

The situation is different when individuals are sampled at different times (serially sampled). In this case, we need to define another kind of phylogenetic tree in which leaves are also ranked.

### Definition 2

***A fully ranked (FR) X‐tree*** *is a pair*(T,h)*, where**is a binary rooted phylogenetic X‐tree andh*:*V*→{1,…,*l*} *with*$\left|\overset{\circ }{V}|<l\leq |V\right|$*is a surjective function such that*

● *v*_1_≤_*T*_*v*_2_ implies *h*(*v*_1_)≤*h*(*v*_2_) and

● *h*(*v*_1_)=*h*(*v*_2_) implies *v*_1_=*v*_2_ or $v_{1},v_{2}\in V\setminus \overset{\circ }{V}$.

**Figure 2:**
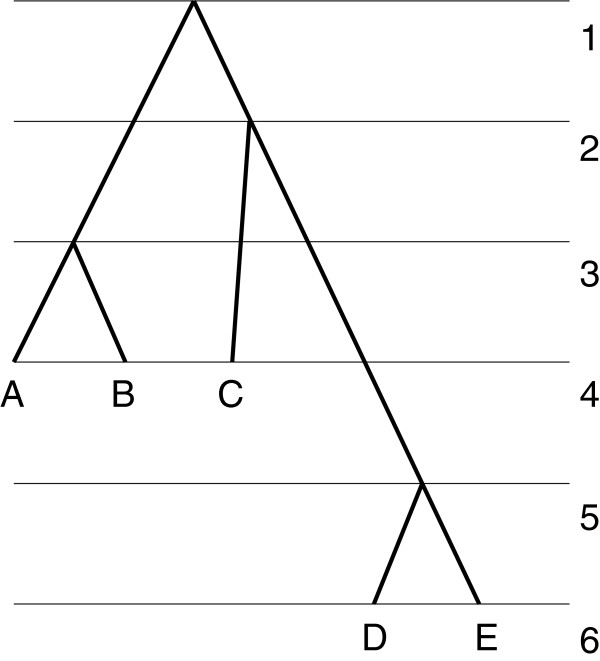
**Fully ranked tree.** Fully ranked *X*‐tree. *X*={*A*,*B*,*C*,*D*,*E*}. The numbers on the right are values of the ranking function.

An example of a fully ranked *X*‐tree is given in Figure 2.

Before the tree is reconstructed we observe only leaves (sampled individuals) of the tree that are grouped (pre‐ranked) according to the times they were sampled. For the tree shown in Figure 2, we have two sampling times and hence two groups: *A*, *B*, and *C* form the first group, *D* and *E* form the second group.

Let T=(T,h) be a fully ranked *X*‐tree with *h*:*V*→{1,…,*l*}. Let *m*=|*h*(*ϕ*(*X*))|, that is, the number of sampling times. Define *a pre‐ranking function* ĥ from *X* onto {1,…,*m*} for tree  T such that for all *x*_1_,*x*_2_∈*X*

● *h*(*ϕ*(*x*_1_))≤*h*(*ϕ*(*x*_2_)) implies $ĥ(x_{1})\leq ĥ(x_{2})$ and

● *h*(*ϕ*(*x*_1_))=*h*(*ϕ*(*x*_2_)) iff $ĥ(x_{1})=ĥ(x_{2})$.

For the tree given in Figure 1, ĥ(A)=ĥ(B)=ĥ(C)=1 and ĥ(D)=ĥ(E)=2.

Let *X* and ĥ:X→{1,…,m} be fixed. We are interested in the number of all fully ranked *X*‐trees that have ĥ as a pre‐ranking function. Note that this number depends only on the numbers $n_{i}=|\{x|ĥ(x)=i\}|$, the number of individuals sampled at the *i*th time point, not on *X* and  ĥ directly. We denote this quantity by *F*(*n*_1_,…,*n*_*m*_), where *F* stands for “fully ranked”. Then

(1) $$F(n_{1},\ldots ,n_{m})=\overset{n_{m}}{\underset{i=1}{\sum }}\frac{R(n_{m})}{R(i)}F(n_{1},\ldots ,n_{m-1}+i)$$

and *F*(*n*)=*R*(*n*).

### Proof

Consider a continuous process of bifurcation in which lineages may bifurcate in time or be cut and labeled (sampled). The process finishes when all lineages are cut producing a tree. The discrete structure of the tree produced by this process is a fully ranked *X*‐tree. It is easy to see that every fully ranked *X*‐tree can be obtained as a result of this process. To count the required number we can count the number of different trees which can be produced by the process if we know that after it finishes there are *n*_*i*_ sampled individuals (i.e., cut and labeled lineages) at the *i*th time point, i.e., we have the sequence (*n*_1_,…,*n*_*m*_).

Suppose that at the (*m*−1)th time point there are *i* lineages that are ancestral to *n*_*m*_ individuals sampled at time *m*. When we look at this process backwards in time the bifurcation events become coalescence events. The number of different ways these *n*_*m*_ lineages coalesce to *i* lineages is $\frac{R(n_{m})}{R(i)}$. This is the number of all possible ranked *X*‐trees on *n*_*m*_ individuals but since we are not interested in the structure of the coalescent after we reach *i* lineages, it is divided by the number of ways in which the remaining *i* lineages can coalesce. Note that if coalescence patterns are different between the (*m*−1)th and *m*th time points then the trees are also different.

Further, for each of these coalescence patterns, we need to count the number of different ways these *i* lineages and other *n*_1_,…,*n*_*m*−1_ lineages can coalesce. This is where we can apply the recursion. We can consider that we also cut these *i* lineages at time *m*−1 and label them with the ranked subtrees descendant from these lineages. Then, at time *m*−1, we have *n*_*m*−1_ sampled individuals and another *i* sampled individuals and it remains to count the number of trees on the sequence (*n*_1_,…,*n*_*m*−1_+*i*).

Note that two trees are different if they have different numbers of lineages at time *m*−1. The number of additional *i* lineages can be between 1 and *n*_*m*_ and we need to sum over all possible *i* to complete the recursion. □

We introduce a third type of tree in which sampled individuals may be direct ancestors of later sampled individuals. We call it a tree with sampled ancestors. This type of tree is not usually considered in phylogenetics since the probability of sampling a direct ancestor is often negligible. In small populations or when a large portion of the population is sampled, however, this can not be ignored.

Let *T*=(*V*,*E*,*ρ*) be a weak binary tree. Define a set $\hat{V}$ as follows:

$$\;\hat{V}\;=\;\{v\;\in \;V|\text{deg}(v)\;=\;1\;\text{or}\;[\;\text{deg}(v)=2\;\text{and}\;v\;\text{is not the root}]\;\}$$

*A rooted S‐phylogenetic X‐tree* is a pair T=(T,ϕ), where *T* is a weak binary tree and $\phi :X\to \hat{V}$ is a bijection.

### Definition 3

***A fully ranked X‐tree with sampled ancestors (FRS X‐tree)*** *is a pair *(T,h)*, where **is a rooted S‐phylogenetic X‐tree andh*:*V*→{1,…,*l*} *is a surjective function such that*

● *v*_1_<_*T*_*v*_2_ implies *h*(*v*_1_)<*h*(*v*_2_) and

● *h*(*v*_1_)=*h*(*v*_2_) implies *v*_1_=*v*_2_ or $v_{1},v_{2}\in \hat{V}$,

(see Figure 3).

**Figure 3:**
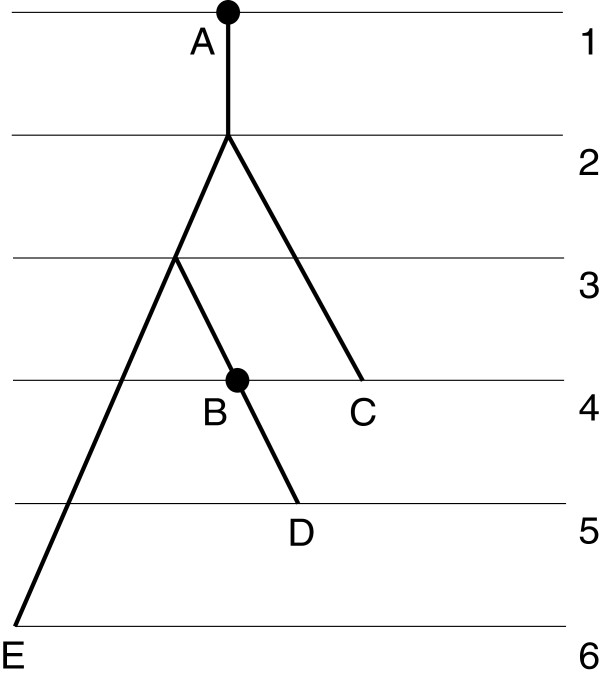
**FRS tree.** FRS *X*‐tree with the labeled 1‐degree root. *X*={*A*,*B*,*C*,*D*,*E*}. The numbers on the right are values of the ranking function.

The definition of a pre‐ranking function remains the same for FRS trees. Let *S*(*n*_1_,…,*n*_*m*_) (with *S* standing for ‘sampled ancestors’) denote the number of all FRS *X*‐trees that have the same pre‐ranking function  ĥ, where $n_{i}=|\{x\;|\;ĥ(x)=i\}|$. Then

(2) S(n1,…,nm)=∑i=1nm∑j=0min{i,nm−1}ijnm−1j×R(nm)R(i)S(n1,…,nm−1+i−j)

and *S*(*n*)=*R*(*n*).

### Proof

Consider the same process as before with only change of sampling events. Now, at some points of time, some lineages are cut and labeled and others are only labeled but not cut.

Then this equation can be obtained as follows. We have *n*_*m*_ individuals that are sampled at the *m*th time point. At time *m*−1, there are between 1 and *n*_*m*_ ancestral lineages of those *n*_*m*_ individuals, depending on the number of coalescence between times *m* and *m*−1. Let *i* denote the number of these lineages. Then there are $\frac{R(n_{m})}{R(i)}$ different possible coalescent patterns that can lead to this situation. Some of these *i* ancestral lineages may be among the individuals sampled at time *m*−1, i.e. lineages that are labeled but not cut at time *m*−1. Let *j* be the number of those ancestral lineages that are sampled at time *m*−1. There are ij ways to chose these *j* lineages out of *i* and there are nm−1j possible ways to chose *j* sampled at time *m*−1 individuals that are not cut at time *m*−1.

Further, at time *m*−1, there are *n*_*m*−1_ sampled lineages and *i*−*j* ancestral lineages that are not sampled and it remains to count the number of FRS trees on the sequence (*n*_1_,…,*n*_*m*−1_+*i*−*j*).

Finally, we sum over all possible *i* and *j* to complete the recursion. □

### Dynamic counting

Calculating the recursions (1) and (2) directly is inefficient and impractical. Here we describe a more efficient algorithm for counting fully ranked trees using these recursions. Rewrite equation (1) as

$$F(n_{1},\ldots ,n_{m})=R(n_{m})\overset{n_{m}}{\underset{i=1}{\sum }}\frac{F(n_{1},\ldots ,n_{m-1}+i)}{R(i)}$$

Then instead of calculating *F*(*n*_1_,…,*n*_*j*_+*α*) for *j*∈{1,…,*m*−1} and *α*∈{0,…,*n*_*j*+1_+…+*n*_*m*_} we can calculate

(3) $$A^{j}(\alpha )=\frac{F(n_{1},\ldots ,n_{j}+\alpha )}{R(\alpha )}$$

using recurrence equations:

(4) $$\begin{array}{ll} & A^{j}(0)=R(n_{j})\overset{n_{j}}{\underset{i=1}{\sum }}A^{j-1}(i)\;\;\text{and}\; \end{array}$$

(5) $$\begin{array}{lll} & A^{j}(\alpha +1)=\frac{\left(n_{j}+\alpha )(n_{j}+\alpha +1\right)}{\alpha (\alpha +1)}A^{j}(\alpha )\; & \;\\ & \;\;\;+\frac{R(n_{j}+\alpha +1)}{R(\alpha +1)}A^{j-1}(n_{j}+\alpha +1).\; \end{array}$$

This leads to Algorithm 1 to calculate *F*(*n*_1_,…,*n*_*m*_). Let *n* be the number of sampled individuals, i.e., $n=\overset{m}{\underset{i=1}{\sum }}n_{i}$. Calculation of all the *R*(*i*) takes *O*(*n*) steps and calculation of all the *A*^*j*^ takes at most *O*(*n*) steps. In total, the algorithm takes *O*(*mn*) steps.

#### Algorithm 1 **Calculating the number of fully ranked trees**

A similar approach leads to an *O*(*mn*^2^)‐time algorithm for counting FRS trees. The description of this algorithm is in Appendix Appendix 1: Algorithm for counting FRS trees.

## Constraints

In phylogenetic analysis, it is common to have some limited information about the ancestors of sampled individuals. We consider two types of such information. First, we may know that a subgroup of sampled individuals is monophyletic. That means that the most recent common ancestor of the subgroup is not an ancestor of any other individual that does not belong to the subgroup. Second, we may know the relative ages of the most recent common ancestors of monophyletic subgroups. This known information imposes constraints on the space of possible phylogenetic trees representing the evolutionary history of sampled individuals. The question is how many phylogenetic trees satisfy the constraints on a group of sampled individuals?

### The number of resolutions of a constraint tree

We first describe a problem for contemporaneous sampling in terms of constraint trees. We call a rooted tree multifurcated if each interior node has at least 2 children. Note that in contrast to the common terminology we assume that a binary tree is also multifurcated. If we replace the word “binar” with “multifurcated” in the definition of a ranked tree we obtain a more general class of trees.

#### Definition 4

***A constraint X‐tree*** *is a multifurcated ranked phylogenetic X‐tree.*

**Figure 4:**
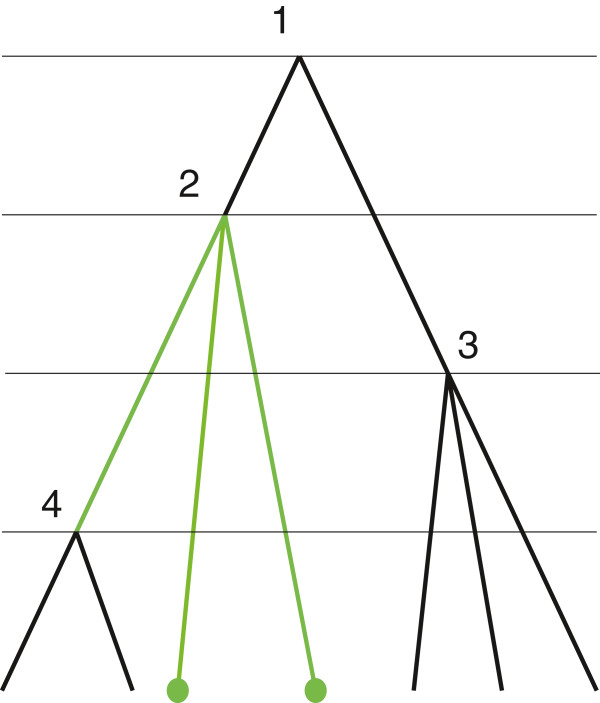
**Constraint tree.** Constraint tree, labels are omitted. Subtree 2 is coloured green. It has two child nodes that are leaves, therefore, n_*2*_*=2. The ancestor function for this tree is defined as f(2)=f(3)=1 and f(4)=2. A compact notation for this constraint tree is *$\left(n_{1},\ldots ,n_{k},f)=(0,2,3,2,\{(2,1),(3,1),(4,2)\}\right)$.

An example of a constraint tree is given in Figure 4. A constraint tree represents prior information about clades and ranking. Each interior node constrains a subgroup of individuals, leaves that are descendant from this node, to by monophyletic. The most recent common ancestor of the whole group of individuals, the root node, is also regarded as a constraint. The ranking function constrains the ages of the most recent common ancestors of the monophyletic subgroups to have a specified order.

We say that a ranked X‐tree $T_{1}=(T_{1},h_{1})$*resolves* a constraint X‐tree $T_{2}=(T_{2},h_{2})$ if there is an isomorphic embedding of $T_{2}$ into $T_{1}$, i.e., there is an injective mapping *f:V*_*2*_*→V*_*1*_ such that 

● ϕ_*1*_*(x)=f(ϕ*_*2*_(*x*)) for each x∈X,

● 

$$v\leq_{T_{2}}u$$

iff $f(v)\leq_{T_{1}}f(u)$ for each u,v∈V_*2*_, and

● *h*_*2*_*(v)≤h*_*2*_(*u*) implies *h*_*1*_*(f(v))≤h*_*1*_*(f(u))* for each $u,v\in \overset{\circ }{\underset{2}{V}}$.

We wish to calculate the number of ranked trees that resolve a given constraint tree T=(T,ϕ,h). It is easy to see that this number depends only on the underlying tree *T* and ranking function *h*, but does not depend on the labeling function ϕ or the label set ***X***.

We now introduce some notation in order to define recursive equations. We label interior nodes according to their ranks such that node i is a node v such that h(v)=i. A subtree induced by node i and its children is called subtree i. Child nodes in a subtree may be leaves in the initial tree. Let *n*_*i*_≥0 denote the number of such child nodes in subtree *i*. Let *f*:{2,…,*k*}→{1,…*k*−1} be the parent function on interior nodes, i.e., *f(i)=j* whenever *j* is a parent to *i*. When *k=1*, i.e., there is only one interior node, *f=∅*. See Figure 4 for an example of introduced notation. Note that a tuple (*n*_*1*_,…,*n*_*k*_*,f*) completely defines a pair (T,h).

Let *R*^*r*^*(n*_*1*_*,…,n*_*k*_*,f*) be the number of ranked trees resolving a constraint tree defined by the tuple (*n*_*1*_*,…,n*_*k*_*,f*). The superscript r stands for “resolution”. Then the following equations hold.

(6) $$\;R^{r}(2,\emptyset )=1,$$

(7) Rr(n1,…,nk−1,2,f)=∑i∈Cni2Rr(n1,…,ni−1,…,nk−1,2,f)+Rr(n1,…,nf(k)+1,…,nk−1,f|{2,…,k−1})and

(8) Rr(n1,…,nk,f)=∑i∈Cni2Rr(n2,…,ni−1,…,nk,f),ifnk>2,

where *C* is the collection of nodes that have more than 2 children and at least 2 of them are leaves, i.e., *C={i<k | n*_*i*_≥2 and *n*_*i*_*+α*_*i*_>2} and *α*_*i*_*=|{a| f(a)=i}|*. Note that *n*_*i*_*+α*_*i*_ is the number of children of node *i*.

#### Proof

**Figure 5:**
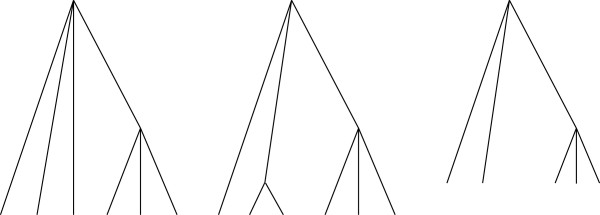
Recursive approach. The last interior node in a resolving tree locates in subtree 2.

When a constraint tree has 2 leaves, it is unique and is resolution of itself. So Equation (6) is trivial. To explain the main sum in Equation (7) and (8) we consider the constraint tree which is defined by (3,3,{(2,1)}) and shown in Figure 5, left. The last interior node of a resolving tree (that is, the interior node with the highest rank or the furthest node from the root) is either a parent to leaves in subtree 1 or leaves in subtree 2. Suppose it is the first case (see Figure 5, centre). Since leaves have distinct labels from *X*, there are 32 ways to chose two leaves that are children of that last node. We can partition all the resolving trees for which the last node is in subtree 1 in 32 groups. The number of trees in each group is the number of trees that resolve a constraint tree defined by (2,3,{(2,1)}) and shown on the right of Figure 5. A similar argument holds if the last node in a resolving tree is a parent to nodes from subtree 2.

So in the general case, there are *k* subtrees and if the last node is in subtree *i* we have ni2 ways to choose two lineages that coalesce and then we should count the number of trees resolving the tree defined by (*n*_*1*_,…,*n*_*i*_*−1,…,n*_*k*_*,f*). Note that in the example above, we consider the tree with more than 2 leaves in each subtree. However, the last interior node of a resolving tree can not be in subtree *i* for *i<k* if there is not enough leaves in this subtree. This can happen either if there are less than 2 leaves in subtree *i* or if there are 2 leaves in subtree *i* and node *i* has only these two leaves as its children. Both cases imply that any parent to leaves of subtree *i* in a resolving tree has a lesser rank than the rank of node *k*. This explains why we sum only over the elements of the set *C*.

Finally, we should consider one more case which explains why there is one more summand in equation (7). If the last node in a constraint tree has only two children, i.e., there are 2 leaves in subtree *k*, then there is one more group of resolving trees, the group that consists of resolving trees that have this node as the last node. □

#### Dynamic counting

We will calculate *R*^*r*^*(n*_*1*_*,…,n*_*k*_*,f*) for the corresponding constraint tree. In order to find *R*^*r*^*(n*_*1*_*,…,n*_*k*_*,f*), at each step s, we will calculate numbers $R^{r}(x_{1},\ldots ,x_{t},f{|}_{t_{-1}})$ with $\underset{i\leq t}{\sum }x_{i}=s$,** t**_*−1*_={2,…,*t*}, and *t≤k*. Note that we do not have to calculate all such numbers. To determine which numbers are required we define two upper triangular matrices m and M of size *k×k*.

**Figure 6:**
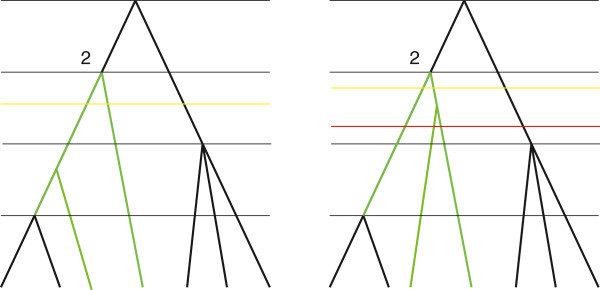
**Defining matrices **m** and**M**.** Two trees that resolve a constraint tree from Figure 4 (only resolutions of subtree 2 are shown) and three ways to draw a horizontal line. The yellow lines correspond to the minimal number of intersections and the red line, to maximal. Thus, m_*2,2*_=2 and M_*2,2*_=3. We can also note that m_*1,2*_=M_*1,2*_=1.

Suppose we draw a horizontal line which is strictly below the line that passes through node *j* and strictly above the line that passes through node *j+1* (or all the leaves if *j=k*). Then m_*i,j*_ is the minimal possible number of intersections of this line with branches of subtree *i* in a resolving tree and M_*i,j*_ is the maximal possible number. An example is given in Figure 6.

Let *a*_*i,j*_*=|{x≤j ∣ f(x)=i}|* for *i≤j*. So *a*_*i,j*_ is the number of children of node *i* with ranks at most *j*. Then

$$\begin{array}{lll} & M_{i,j}=n_{i}+\alpha_{i}-a_{i,j}\;\;\text{and}\; & \;\\ & m_{i,j}=\left\{\begin{array}{l} 2\;\;\text{if}\;\;a_{i,j}=0,\\ 1\;\;\text{if}\;\;a_{i,j}>0\;\;\text{and}\;\;M_{i,j}>0,\\ 0\;\;\text{otherwise.} \end{array}\right)\; & \; \end{array}$$

Let *t≤k* and $x_{1},\ldots ,x_{t}\in N$. We call a tuple $\left(x_{1},\ldots ,x_{t},f{|}_{t_{-1}}\right)$ eligible if m_*i,t*_*≤x*_*i*_≤M_*i,t*_ for 1≤*i*≤*t*.

We now turn to Algorithm 2 to count resolutions. At each step *s≤n*, we construct a set *S*_*s*_. A unique element of *S*_*n*_ is *R*^*r*^*(n*_*1*_*,…,n*_*k*_*,f*) and calculating elements of *S*_*s*_ only requires elements of *S*_*s−1*_.

##### Algorithm 2 Calculating the number of resolutions of a constraint tree

##### Proposition 1

*When k is fixed Algorithm* 2 *does at most O(n*^*k*^*) steps.*

##### Proof

The algorithm does *O(k)* steps for each eligible tuple and, since we assume *k* is a constant, it is *O*(1). For given *j* there are

$$\overset{j}{\underset{i=1}{\prod }}(M_{i,j}-m_{i,j}+1)$$

eligible tuples of size *j*. Since M_*i,j*_−m_*i,j*_*+1≤n*_*i*_*+α*_*i*_−1, for the total number of eligible tuples we have

$$\begin{array}{l} \;\overset{k}{\underset{j=1}{\sum }}\;\overset{j}{\underset{i=1}{\prod }}(M_{i,j}\;-\;m_{i,j}\;+\;1)\;\leq \;\overset{k}{\underset{i=1}{\sum }}(n_{1}\;+\;\alpha_{1}\;-\;1)\;\cdot \ldots \cdot \;(n_{i}\;+\;\alpha_{i}-1)\;<\\ \;k(n_{1}\;+\;k\;-\;1)\;\cdot \ldots \cdot \;(n_{k}\;+\;k\;-\;1)\;\leq \;k{\left(\frac{n}{k}\;+\;k\;-\;1\right)}^{k}\;=\;O(n^{k}) \end{array}$$

□

### The number of resolutions of a fully ranked constraint tree

We can generalise the results of the previous section to fully ranked trees. Now we replace the word “binary” with “multifurcated” in the definition of a fully ranked tree to get

#### Definition 5

***A fully ranked constraint X‐tree*** *is a pair *(T,h)*, where** is a multifurcated rooted phylogenetic X‐tree and h is a function such that h:V→{1,…,l}, where *$\left|\overset{\circ }{V}|<l\leq |V\right|$*, and*

● *v*_*1*_*≤*_*T*_*v*_*2*_* implies h(v*_*1*_*)≤h(v*_*2*_*),*

● *h(v*_*1*_*)=h(v*_*2*_*) implies v*_*1*_*=v*_*2*_* or *$v_{1},v_{2}\in V\setminus \overset{\circ }{V}$.

We say that a fully ranked X‐tree $T_{1}=(T_{1},h_{1})$*resolves* a fully ranked constraint X‐tree $T_{2}=(T_{2},h_{2})$ if there is an isomorphic embedding of $T_{2}$ into $T_{1}$, i.e., there is an injective mapping *f:V*_*2*_*→V*_*1*_ such that 

● *ϕ*_*1*_*(x)=f(ϕ*_*2*_*(x))* for each x∈X,

● 

$$v\leq_{T_{2}}u$$

iff $f(v)\leq_{T_{1}}f(u)$ for each *u,v∈V*_*2*_*,*

● *h*_*2*_*(v)≤h*_*2*_*(u)* implies *h*_*1*_*(f(v))≤h*_*1*_*(f(u))* for each *u,v∈V*_*2*_, and

● *h*_*2*_*(v)=h*_*2*_*(u)* iff *h*_*1*_*(f(v))=h*_*1*_*(f(u))* for each *u,v∈V*_*2*_.

The problem is to count the number of fully ranked resolutions of a fully ranked constraint tree.

Let T=(T,h) be a fully ranked constraint tree with h:V→{1,…,l} and$|\overset{\circ }{V}|=k$. Again we label interior nodes with numbers from {1,…,k}. However, labels and ranks of interior nodes do not necessary coincide now. Let $r:\{1,\ldots ,k\}\to h(\overset{\circ }{V})$ be an injective increasing function which maps the kth interior node to its rank. Note that r(1) is always equal to 1 because the root node always has rank 1 and that r(k)<l because only leaves can have rank l. Now, node i is the node that has rank r(i) and subtree i is the subtree which is induced by node i and its children. Since leaves in subtrees are now ranked, we need more parameters to encode the tree. Let **n**= [ *n*_*i*,*j*_] be a matrix of size*k*×*l*, where*n*_*i*,*j*_ is the number of leaves of rank*j* in subtree*i* or the number of children of node*i* with rank*j*. Note that if node*i* is a parent of node*j* then*n*_*i*,*r*(*j*)_=1 and*n*_*i*,*x*_=0 for*x***≠***r*(*j*) and this means that the parent function is uniquely defined by the matrix **n** and function*r* and the number of resolutions of T depends only on (**n**,*r*).

If a constraint tree has only 2 leaves then there is only 1 tree resolving it:

$$F^{r}((2),r_{0})=F^{r}((1,1),r_{0})=1$$

with*r*_*0*_ mapping 1 to 1.

For the main recursion we need to determine the location of the last interior node in a resolving tree. As before, this node can be a parent to leaves in different subtrees. Also, since leaves now may have different ranks, the last interior node in a resolving tree may be ranked in different ways with respect to ranks of the leaves. Let*N*_*c*,*x*_ denote the number of children of node*c* with ranks at least*x*, that is, $N_{c,x}=\overset{l}{\underset{j=x}{\sum }}n_{c,j}$. For each*p*∈{*r*(***k***)+1,…,*l*}, we define a set*C*^*p*^ of candidate subtrees in which the last node can be placed at level*p*, i.e. between the (*p*−1)th and*p*th time points.

$$C^{p}=\{c\;|\;N_{c,p}\geq 2\;\;\text{and}\;\;N_{c,r(c)+1}>2\}$$

**Figure 7:**
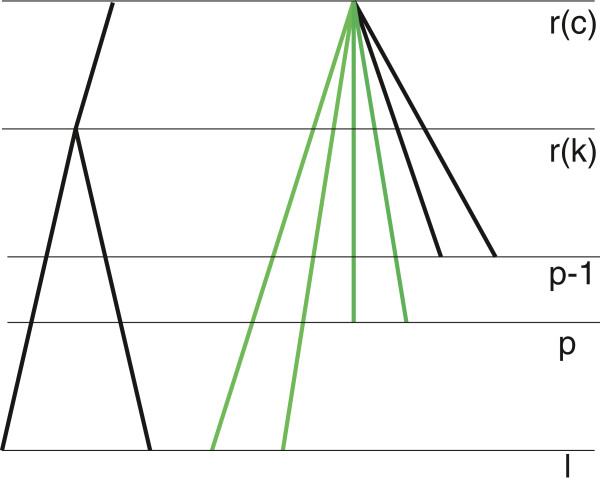
Location of a new node between the (*p*−1)‐th and*p*‐th time points. A new node can be placed in subtree*c* between the (*p*−1)‐th and*p*‐th time points if the number of green branches is greater or equal to 2 and the number of all branches in subtree*c* is greater than 2.

See Figure 7 for an example of subtree where the last node can be placed between the (*p*−1)th and*p*th time points.

For each*p* and*c* such that*c*∈*C*^*p*^, there is a distinct group of resolving trees. The quantity of trees in this group is equal to the number of resolutions of a constraint tree which is defined by matrix $n^{c,p}=\;[\;n_{i,j}^{c,p}]$ of size*k*×*p* such that 

– 

$$n_{i,j}^{c,p}=n_{i,j}$$

for 1≤*i*≤*k* and*j*<*p*,

– 

$$n_{c,p}^{c,p}=N_{c,p}-1$$,

– 

$$n_{i,p}^{c,p}=N_{i,p}$$

for*i*≤*k* and*i*≠*c*.

If node*k* has only 2 children then, as before, there is one more group of resolving trees. This group consists of the resolving trees in which the last interior node coincides with node*k*. Let$n^{\prime }=\;[\;n_{i,j}^{\prime }]$ be a matrix of size (*k*−1)×*r*(*k*) such that 

– 

$$n_{i,j}^{\prime }=n_{i,j}$$

for 1≤*i*≤*k* and*j*<*r*(*k*),

– 

$$n_{i,r(k)}^{\prime }=N_{i,r(k)}$$

for 1≤*i*≤*k*−1.

This matrix defines a constraint tree for which the number of resolutions is equal to the number of trees in the last group.

Then the main recursion is as follows:

(9) Fr(n,r)=∑p=r(k)+1l∑c∈CpNc,p2Fr(nc,p,r)+Fr(n′,r|{1,…,k−1})ifNk,r(k)+1=2,

(10) Fr(n,r)=∑p=r(k)+1l∑c∈CpNc,p2Fr(nc,p,r)otherwise,

Using these equations, we can calculate*F*^*r*^(**n**,*r*) for*O*(*m*^*2*^*n*^k^) steps, where*m* is the number of sampling points, i.e.,*m*=*l*−*k*. The calculation is described in Appendix Appendix 2: Algorithm for counting fully ranked resolutions of a fully ranked constraint tree.

## Conclusions

In Table 1, we summarised the complexity (upper bounds) of the counting algorithms. We can see that counting fully ranked and FRS trees is reasonably fast, particularly when the number of sampling points is small. Counting of resolutions of a constraint tree is an expensive procedure but for small*k* it remains possible. In practice,*k* is typically small so this algorithm will be of practical use. Counting resolutions of an FRS constraint tree, using the same methods, has a greater cost and is less elegant, but its practicality and bounds on its complexity remain to be assessed.

**Table 1:** The complexity of algorithms

	**No constraints** **( *k *** **=1)**	***k* constraints**
Contemporaneous		
sampling	*O*(*n*)	*O*(*n*^*k*^)
(*m*=1)		
Serial sampling		
with no sampled	*O*(*mn*)	*O*(*m*^*2*^*n*^*k*^)
ancestors		
Serial sampling		
with sampled	*O*(*mn*^*2*^)	‐
ancestors		

The table summaries the complexity of the counting algorithms, where*n*** is the sample size,***k*** is the number of constraints, and***m*** is the number of sampling time‐points. We assume that***k* is fixed.

These algorithms can be implemented in software for phylogenetic analysis that involves serial sampling scheme or limited prior knowledge about ancestors of particular clades for calculating tree prior distributions.

## Appendix 1: Algorithm for counting FRS trees

Rewrite equation (2) as follows:

$$S(n_{1},\ldots ,n_{m})=\overset{n_{m}}{\underset{i=0}{\sum }}A(i,n_{m-1},n_{m})S(n_{1},\ldots ,n_{m-1}+i)$$

where A(i,a,b)=∑x=0min{a,b−i}R(b)R(i+x)i+xxax. Then we can calculate*S*(*n*_*1*_*,…,n*_*m*_) recursively using Algorithm 3. We use the notation $N_{j}=\overset{m}{\underset{i=j}{\sum }}n_{i}$.

### Algorithm 3 **Calculating the number of FRS trees**

At each step*j*>1 of the algorithm, we calculate*S*(*n*_*1*_*,…,n*_*j*_+*α*) for 0≤*α*≤*N*_*j*+1_. We skip step*j*=1 and do not calculate*S***(*n***_*1*_*+α*) for 0≤*α***≤***N*_*2*_ because*S***(***x***)=***R***(***x*) and we have already calculated all the necessary*R*(*x*). Further, for calculating each*S*(*n*_*1*_*,…,n*_*j*_**+***α*), we need to calculate the coefficients*A* and this is the most expensive part of the algorithm. So, at each step ***j*** >1, we need to calculate*A***(***i***,** ***n***_***j***−1_,*n*_*j*_**+***α*) for 0≤*i***≤***n*_*j*_**+***α* and 0≤*α***≤***N*_*j*+1_.

First, we calculate these values for*α*=0. Denote

B(i,a,b,x)=R(b)R(i+x)i+xxax,forx≤aandi+x≤b

then

$$A(i,a,b)=\overset{\text{min}\{a,b-i\}}{\underset{x=0}{\sum }}B(i,a,b,x)$$

where 0≤*i***≤***b*,*a*=*n*_*j*−1_, and*b***=***n*_*j*_. When*i* is fixed, calculation of*A***(***i*,*n*_*j*−1_,*n*_*j*_) requires values of*B*(*i*,*n*_*j*−1_, ***n***_***j***_, ***x***) for 0≤ ***x*** **≤** ***m*** ***i*** ***n*** **{***n*_*j*−1_,*n*_*j*_−***i***}. Rewrite this as*B***(***n*_*j*_−*β*,*n*_*j*−1_,*n*_*j*_,*x*) for 0≤*x***≤***min*{*n*_*j*−1_,*β*} with*β*= ***n***_*j*_−*i*. Now we can calculate these values for 0≤*β*≤*n*_*j*_ and, hence, for 0≤*i***≤***n*_*j*_ using recursive equations:

() $$\;B(n_{j},n_{j-1},n_{j},0)=1$$

(11) $$\begin{array}{lll} \;B(n_{j} & -(\beta +1),n_{j-1},n_{j},x)\; & \;\\ & =\frac{\left(n_{j}-(\beta +1)+x)(n_{j}-\beta )B(n_{j}-\beta ,n_{j-1},n_{j},x\right)}{2}\; \end{array}$$

(12) $$\begin{array}{lll} \;B(n_{j} & -(\beta +1),n_{j-1},n_{j},(\beta +1))\; & \;\\ & =\frac{\left(n_{j}-\beta )(n_{j-1}-\beta )B(n_{j}-\beta ,n_{j-1},n_{j},\beta \right)}{{\left(\beta +1\right)}^{2}}\; \end{array}$$

We use equation (11) for 0≤*x*≤*min***{*n***_***j***−1_, ***β***}. If ***m*** ***i*** ***n*** {***n***_***j***−1_, ***β*** +1}≠ ***m*** ***i*** ***n*** **{*n***_***j***−1_, ***β***} and therefore ***m*** ***i*** ***n*** **{*n***_***j***−1_, ***β*** +1}= ***m*** ***i*** ***n*** **{*n***_***j***−1_, ***β***}+1= ***β*** +1 then we also use equation (12). The cost of this calculation is dominated by the number of the summands ***B*** **(*n***_***j***_**−*β***, ***n***_***j***−1_, ***n***_***j***_, ***x***) for 0≤ ***x*** **≤** ***m*** ***i*** ***n*** **{*n***_***j***−1_, ***β***} and 0≤ ***β*** **≤** ***n***_***j***_. This number is $O(n_{j}^{2})$.

Now we need to calculate ***A*** (0, ***n***_***j***−1_, ***n***_***j***_+ ***α***),…, ***A*** (***n***_***j***_**+** ***α***, ***n***_***j***−1_, ***n***_***j***_**+** ***α***) for 1≤ ***α*** **≤** ***N***_***j*** +1_. Having ***A*** (0, ***n***_***j***−1_, ***n***_***j***_+ ***α***),…, ***A*** (***n***_***j***_+ ***α***, ***n***_***j***−1_, ***n***_***j***_+ ***α***) calculated (note that we have already calculated these values for ***α*** =0), we can calculate ***A*** (0, ***n***_***j***−1_, ***n***_***j***_+(***α*** +1)),…, ***A*** (***n***_***j***_+(***α*** +1), ***n***_***j***−1_, ***n***_***j***_+(***α*** +1)) using equations

(13) A(i,a,b+1)=(b+1)b2A(i,a,b)+b+1iab+1−iifb−i<a,(b+1)b2A(i,a,b)ifa≤b−iandA(b+1,a,b+1)=1

where 0≤ ***i*** **≤** ***b***, ***a*** **=** ***n***_***j***−1_, and ***b*** = ***n***_***j***_+ ***α***.

Moreover, for each ***α***, we can optimize calculation of the second summands in the first case of equation (13) using recursion

(14) nj+αi+1nj−1nj+α−(i+1)=(nj+α−i)2(i+1)(nj−1−nj−α+i+1)nj+αinj−1nj+α−i

To apply this recursion we need an initial value. This value depends on ***n***_***j***−1_, ***n***_***j***_, and ***α*** and, since only the first case of equation (13) contains the second summand, it is not necessary the value of this summand for ***i*** =0. There are 3 cases for calculation of all the initial values that are necessary at step ***j***. 

Case 1: ***N***_***j***_**<** ***n***_***j***−1_. That means that we use the first case of equation (13) for all ***α***. In this case, for all ***α***, the initial value for recursion (14) is the value of the second summand in (13) when ***i*** =0. So we need to calculate nj+α0nj−1nj+α+0 for 1≤ ***α*** **≤** ***N***_***j*** +1_ and those are nj−1nj+1,…,nj−1nj+Nj+1. This takes ***O*** (***N***_***j***_) steps.

Case 2: ***n***_***j***_+ ***α***_***0***_ ***=*** ***n***_***j***−1_ for some ***α***_***0***_ ***∈{0,…,*** ***N***_***j*** +1_}. If ***α***_***0***_>0 then, for all ***α*** **≤** ***α***_***0***_, we use the first case of (13) and therefore we need to calculate nj−1nj+1,…,nj−1nj+α0, this takes ***O*** **(*α***_***0***_) steps. For all ***α*** **>** ***α***_***0***_, we use the second case first and, starting from ***i*** **=** ***α*−*α***_***0***_, we use only the first case. So we need to calculate nj+αα−α0nj−1nj+α−(α−α0) for ***α*** ∈{***α***_***0***_+1,…, ***N***_***j*** +1_}, which arenj−1+11,…,nj−1+(Nj+1−α0)(Nj+1−α0). This takes ***O*** (***N***_***j*** +1_−***α***_***0***_) steps. In total, we have ***O*** **(*N***_***j*** +1_) steps.

Case 3: ***n***_***j***−1_< ***n***_***j***_. That means that, for all ***α***, we use the second case first and, starting from ***i*** **=** ***n***_***j***_**+** ***α*−*n***_***j***−1_, we use only the first case. Calculatenj+1nj−1,…,nj+Nj+1nj−1 for ***O*** **(*n***_***j***−1_+ ***N***_***j*** +1_) steps.

Each case costs at most ***O*** **(*N***_***j***_). Provided that the initial values are calculated, the cost of the rest calculation at step ***j*** is dominated by the number of the coefficients ***A*** **(*i***, ***n***_***j***−1_, ***n***_***j***_**+** ***α***) for 0≤ ***i*** **≤** ***n***_***j***_**+** ***α*** and 1≤ ***α*** **≤** ***N***_***j*** +1_ and it is$O(N_{j}^{2})$. Summing up$O(N_{j}^{2})$X, ***O*** **(*N***_***j***−1_), and $O(N_{j}^{2})$ gives us the cost of each step ***j***, which is $O(N_{j}^{2})$. Since 1< ***j*** **≤** ***m*** and calculation of ***R*** **(*i***) for 0≤ ***i*** **≤** ***N***_***1***_ ***=*** ***n*** takes ***O*** **(*n***), the algorithm does ***O*** **(*m*** ***n***^***2***^) steps in total.

## Appendix 2: Algorithm for counting fully ranked resolutions of a fully ranked constraint tree

The algorithm for counting resolutions of a constraint tree requires a few changes to count resolutions of a fully ranked constraint tree. Recall that, at each step ***s***, we calculated the set ***S***_***s***_ which consists of the numbers of resolutions of intermediate trees with ***s*** leaves. To construct ***S***_***s***_, for each element of ***S***_***s***−1_, we proposed a collection of tuples that may define intermediate trees with ***s*** leaves. To accept eligible tuples we defined two matrices m and M. The general scheme for counting resolutions of a fully ranked constraint tree is the same. However, we need to define matrices m and M for a fully ranked constraint tree and describe the procedure of proposing new tuples. The procedure becomes more technical because of additional ranking.

We will calculate ***S***^***r***^(**n**, ***r***) for the corresponding fully ranked constraint tree  T. Let

$$a_{i,j}=|\{x\;|\;\;\text{node}i\text{is a parent of node}\;\;x\;\;\text{and}\;\;r(x)\leq j\}|$$

for 1≤ ***i*** **≤** ***k*** and ***r*** **(*i*)≤** ***j*** **≤** ***l***−1, i.e., ***a***_***i***, ***j***_ is the number of interior nodes that are children of node ***i*** and have rank at most ***j***. Define two matrices m and M of size ***k*** **×(*l***−1) such that

$$M_{i,j}=N_{i,j+1}\;\text{and}\;m_{i,j}=\left\{\begin{array}{l} 2\;\;\text{if}\;\;a_{i,j}=0,\\ 1\;\;\text{if}\;\;a_{i,j}>0\;\;\text{and}\;\;M_{i,j}>0,\\ 0\;\;\text{otherwise.} \end{array}\right)$$

As before, if we consider a horizontal line that is strictly between the horizontal line that passes through the nodes of rank ***j*** and the horizontal line that passes through the nodes of rank (***j*** +1) then these matrices determine the minimal and maximal possible numbers of intersections of this line with branches of subtree ***i***.

Let **x**= [***x***_***i***, ***j***_] be a matrix of size ***t*** **×** ***q***, where ***t*** **≤** ***k*** and ***q*** **≤** ***l***, and **t**={1,…, ***t***}. A tuple (**x**, ***r*** **|**_t_) is eligible if 

– ***x***_***i*,** ***j***_x= ***n***_***i***, ***j***_ for 1≤ ***i*** **≤** ***t*** and 1≤ ***j*** **<** ***q***, and

– 

$$m_{i,q-1}\leq x_{q}^{i}\leq M_{i,q-1}$$

for 1≤ ***i*** **≤** ***t***.

Having an intermediate tree with ***s***−1 leaves, we need to consider all the possible ways to transform this tree to a tree with ***s*** leaves. First we need to add a new leaf to some subtree. We give the highest rank to this leaf. After that we may add new time points below the last time point. If we add new time points, we should rerank the leaves with the highest rank such that the new tree has enough leaves at each time point. Let **x**= [***x***_***i***, ***j***_] be a matrix of size ***t*** **×** ***q*** for ***t*** **≤** ***k*** and ***q*** **≤** ***l***. This matrix represents an intermediate tree with ***s***−1 leaves. Suppose we add a new leaf to subtree ***i***_***0***_, where 1≤ ***i***_***0***_ ***≤*** ***t***. Let ***q***_***0***_ be the number of time points in a new tree and, therefore, ***q*** **≤** ***q***_***0***_ ***≤*** ***r*** (***t*** +1) (or ***q*** **≤** ***q***_***0***_ ***≤*** ***l*** if ***t*** = ***k***). Define a function*a* ***d*** ***d*** ***o*** ***n*** ***e*** ((**x**,*r*|_**t**_),*i*_*0*_*,q*_*0*_) which returns a tuple $\left(x^{\prime },r{|}_{t}\right)$, where$x^{\prime }=\;[\;x_{i,j}^{\prime }]$ is a matrix of size*t*×*q*_*0*_ such that 

– 

$$x_{i_{0},q_{0}}^{\prime }=x_{i_{0},q}-\sum_{j=q}^{q_{0}-1}n_{i_{0},j}+1$$,

– 

$$x_{i,q_{0}}^{\prime }=x_{i,q}-\sum_{j=q}^{q_{0}-1}n_{i,j}$$

for ***i*** ≠*i*_***0***_,

– 

$$x_{i,j}^{\prime }=x_{i,j}$$

for 1≤*i*≤ ***t*** and*j*<*q*, and

– 

$$x_{i,j}^{\prime }=n_{i,j}$$

for 1≤*i*≤*t* and*q*≤*j*<*q*_*0*_.

If this tuple is eligible then it represents a new intermediate tree with ***s*** leaves.

At each step*s* of the algorithm, for each tuple (**x**,*r***|**_**t**_) such that*F*^*r*^(**x**,*r***|**_**t**_*)∈S*_*s*−1_, we need to propose a collection of new tuples. The first part of this procedure is Algorithm 4. To stop the procedure, when we recognise that a new tree can not have ***x*** time points, we use a predicate*ext*(*x*) which is true if we can extend the number of time points in a new tree to*x*. The number of time points in a new tree can not always be extended to any arbitrary number because there may not be enough leaves of the highest rank to rerank them in such a way that there will be*n*_*i*,*j*_ leaves of each new rank*j*. So*ext*(*q*) is always true because the tree to which we add a new leaf already has*q* time points.

### Algorithm 4 Working with a tuple (**x**,*r*|_**t**_) such that*F*^*r*^(**x**, ***r*** **|**_**t**_)∈ ***S***_*s*−1_ (part 1)

If ***t*** **<***k* then the proposing procedure contains the second part. In this case, we also can increase the number of leaves in an intermediate tree by adding a new subtree. That means that one of the leaves of the tree becomes a parent to two new leaves. Again we may add new time points. Let ***q***_***0***_ be the number of time points in a new tree and, therefore, ***r*** (***t*** +1)< ***q***_***0***_ ***≤*** ***r*** (***t*** +2) (or ***r*** (***t*** +1)< ***q***_x ***0***_ ***≤*** ***l*** when ***t*** +1= ***k***). We define another function ***a*** ***d*** ***d*** ***c*** ***o*** ***n*** ***s*** ***t*** ***r*** ***a*** ***i*** ***n*** ***t*** ((**x**, ***r*** |_**t**_), ***q***_***0***_) which returns a tuple $\left(x^{\prime },r{|}_{t+1}\right)$, where $x^{\prime }=\;[\;x_{i,j}^{\prime }]$ is a matrix of size (***t*** +1)× ***q***_***0***_ such that 

– 

$$x_{t+1,q_{0}}^{\prime }=2-\sum_{j=r(t+1)+1}^{q_{0}-1}n_{t+1,j}$$,

– 

$$x_{t+1,j}^{\prime }=n_{t+1,j}^{\prime }$$

for 1≤ ***j*** < ***q***_***0***_,

– 

$$x_{i,q_{0}}^{\prime }=x_{i,q}-\sum_{j=q}^{q_{0}-1}n_{i,j}$$

for 1≤ ***i*** **≤** ***t***,

– 

$$x_{i,j}^{\prime }=x_{i,j}^{\prime }$$

for 1≤ ***i*** **≤** ***t*** and ***j*** **<** ***q***, and

– 

$$x_{i,j}^{\prime }=n_{i,j}^{\prime }$$

for 1≤ ***i*** **≤** ***t*** and ***q*** **≤** ***j*** **<** ***q***_***0***_.

This tuple defines a new tree if it is eligible. The second part of the procedure is Algorithm 5. The values of ***q***_***0***_ and ***e*** ***x*** ***t*** (***q***_***0***_) are as after running Algorithm 4.

### Algorithm 5 Working with a tuple (**x**, ***r*** **|**_**t**_) such that ***F***^***r***^(**x**, ***r*** **|**_**t**_)∈ ***S***_***s*−1**_ (part 2)

Finally, as before, the main algorithm calculates sets*S*_*s*_ recursively for 0≤*s***≤***n*, where*n* is the number of leaves in a constraint tree  T, and a unique element of*S*_*n*_ is*S*^*r*^(**n**,*r*).

Let ***m*** be a number of sampling points, i.e., ***m*** =*l*−*k*.

### Proposition 2

If*k* is fixed then the algorithm does at most*O*(*m*) steps for each eligible tuple.

### Proof

Let (**x** ***r*** **|**_**t**_) be an eligible tuple. First, having all the necessary summands for equations (9) and (10), we need to calculate ***F***^***r***^(**x**, ***r*** **|**_**t**_). This takes at most ***O*** **(*t*** **×[*r*** (***t*** +1)−***r*** (***t***)]) steps. Since we assume ***k*** is a constant and since ***t*** ≤ ***k*** and ***r*** **(*t*** +1)−***r*** (***t***)≤ ***m***, it is ***O*** (***m***). Second, we need to perform the procedure of proposing new tuples for this tuple. The most expensive part here is calculation of the last columns of matrices $x^{\prime }$ because it involves calculation of the sums $\sum_{j=q}^{q_{0}-1}n_{i,j}$. Note that, storing the values of $x_{i,q}-\sum_{j=q}^{q_{0}-1}n_{i,j}$ for 1≤ ***i*** **≤** ***t*** at each step ***q***_***0***_, we can optimise these calculations. Then it takes ***O*** (***t*** ×(***q*−*r*** **(*t*** +2)) steps and it is ***O*** **(*m***) again. □

### Proposition 3

If k is fixed then the algorithm has O(^m^n^k^) time complexity.

### Proof

Let ***i***_***x***_ be such a number that ***r*** (***i***_***x***_)≤ ***x*** **<** ***r*** (***i***_***x***_+1). Then the number of eligible tuples is

$$\overset{l-1}{\underset{j=1}{\sum }}\overset{i_{j}}{\underset{i=1}{\prod }}(M_{i,j}-m_{i,j}+1)$$

Note that

$$M_{i,j}-M_{i,j}+1\leq N_{i,j}+1\leq N_{i,r(i)+1}+1=b_{i}+1$$

2where ***b***_***1***_ ***+…+*** ***b***_***k***_**=** ***n*** **+** ***k***−1. Then

$$\begin{array}{lll} \overset{l-1}{\underset{j=1}{\sum }}\overset{i_{j}}{\underset{i=1}{\prod }}(M_{i,j}-M_{i,j}+1) & <(l-1)(b_{1}+1)\ldots (b_{k}+1)\; & \;\\ & <(l-1){\left(\frac{n}{k}\right)}^{k}=O(mn^{k})\; & \; \end{array}$$

From this and Proposition 2, it follows that the algorithm does at most*O*(*m*^***2***^ ***n***^***k***^) steps. □

## Competing interests

The authors declare that they have no competing interests.

## Authors’ contributions

The authors equally contributed to conceive the work. AG drafted the manuscript and DW and AJD revise it. All authors read and approved the final manuscript.
